# Exosomal lncRNA H19 promotes the progression of hepatocellular carcinoma treated with Propofol via miR‐520a‐3p/LIMK1 axis

**DOI:** 10.1002/cam4.3313

**Published:** 2020-08-07

**Authors:** Dongmei Wang, Na Xing, Tao Yang, Junqi Liu, Huaping Zhao, Juan He, Yanqiu Ai, Jianjun Yang

**Affiliations:** ^1^ Department of Anesthesiology, Pain and Perioperative Medicine The First Affiliated Hospital of Zhengzhou University Zhengzhou China; ^2^ Department of Anesthesiology The Fifth Affiliated Hospital of Zhengzhou University Zhengzhou China; ^3^ Department of Radiotherapy The First Affiliated Hospital of Zhengzhou University Zhengzhou China

**Keywords:** exosome, H19, hepatocellular carcinoma, LIMK1, miR‐520a‐3p

## Abstract

**Background:**

Hepatocellular carcinoma (HCC) is one of the leading causes of cancer‐related deaths globally. Herein, we explored the underlying mechanism by which Propofol inhibited the development of HCC.

**Methods:**

3‐(4,5‐Dimethylthiazol‐2‐yl)‐2,5‐diphenyltetrazolium bromide (MTT) assay was carried out to detect the viability and proliferation. Quantitative real‐time polymerase chain reaction (qRT‐PCR) and Western blot were performed to detect the expression of long noncoding RNA (lncRNA) H19, microRNA‐520a‐3p (miR‐520a‐3p), LIM domain kinase 1 (LIMK1), metastasis‐associated markers (Snail, Twist, Vimentin and E‐cadherin) and exosome markers (CD9 and CD81). Transmission electron microscopy (TEM) was used to observe the morphology and structure of exosomes. The apoptosis and metastasis were measured by flow cytometry and transwell assays. StarBase software was utilized to predict the targets of H19 and miR‐520a‐3p. Dual‐luciferase reporter assay was performed to confirm the interaction between miR‐520a‐3p and H19 or LIMK1. Nude mice bearing tumors were used to validate the role of exosomal H19.

**RESULTS:**

The high expression of exosomal H19 accelerated the proliferation and motility while hampering the apoptosis of HCC cells. MiR‐520a‐3p could bind with H19. Exosomal H19 exacerbated HCC through sponging miR‐520a‐3p. The 3’ untranslated region (3’UTR) of LIMK1 could bind to miR‐520a‐3p. MiR‐520a‐3p mimic transfection reversed the inhibitory effect of high expression of exosomal LIMK1 on the apoptosis of HCC cells and the promoting effects on the proliferation and metastasis of HCC cells. The mRNA and protein levels of LIMK1 were regulated by H19/miR‐520a‐3p signaling. The high level of exosomal H19 promoted the growth of HCC tumors in vivo.

**Conclusion:**

Circulating H19 promoted the proliferation, migration and invasion and inhibited the apoptosis of HCC cells treated with Propofol through upregulating LIMK1 via sponging miR‐520a‐3p.

## Graphical abstract

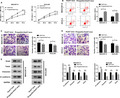

**Highlights**

1. Circulating lncRNA H19 accelerates the proliferation and metastasis and impedes the apoptosis of HCC cells.
2. MiR‐520a‐3p is verified as a target of H19.
3. LIMK1 is a direct downstream gene of miR‐520a‐3p.
4. Exosomal H19 facilitates the malignant potential of Propofol‐exposed HCC cells via miR‐520a‐3p/LIMK1 axis in vivo and in vitro.

## 1 INTRODUCTION

The prognosis of HCC patients remains dismal due to the metastasis and recurrence. Propofol is a common intravenous anesthetic agent. The anti‐tumor role of Propofol has been reported in former articles.^1^, ^2^, ^3^, ^4^ Zhang *et al*. claimed that Propofol inhibited the development of HCC.^5^, ^6^ Ou *et al*. proved that Propofol blocked the proliferation and motility of HCC cells via HMGA2 and Wnt/β‐catenin signaling.^7^ Nevertheless, the potential molecular mechanism by which Propofol exerts its anti‐tumor role in HCC remains to be elucidated.

Exosomes are a group of small membrane vesicles containing proteins, DNA and RNA, and they are derived from multivesicular bodies (MVBs) and eventually release to the extracellular environment.^8^, ^9^ Exosomes have been reported to serve as important modulators in the tumor microenvironment to regulate the development of multiple tumors.^10^, ^11^, ^12^

Long noncoding RNAs (lncRNAs) could act as microRNAs (miRNAs) sponges to function.^13^ The oncogenic role of lncRNA H19 has been reported in multiple cancers. For instance, Lv *et al*. proved that lncRNA H19 accelerated the metastasis of bladder cancer via miR‐29b‐3p.^14^ Yang *et al*. demonstrated that H19 facilitated the motility of colon cancer cells through sponging miR‐138 and upregulating HMGA1.^15^ Bai *et al*. reported that Propofol inhibited the motility of breast cancer through declining the expression of H19.^16^ The aim of our study was to explore the working mechanism of lncRNA H19 in the progression of HCC.

miRNAs are another group of non‐coding RNAs. MiR‐520a‐3p was a tumor suppressor in a variety of cancers. Li *et al*. proved that miR‐520a‐3p blocked the development of breast cancer via CCND1 and CD44.^17^ Qu *et al*. reported that lidocaine could restrain the growth and promote the apoptosis of colorectal cancer cells through elevating the level of miR‐520a‐3p.^18^ Wang *et al*. proved that dexmedetomidine hampered the proliferation and motility while accelerated the apoptosis of osteosarcoma cells through upregulating miR‐520a‐3p.^19^ In this study, we intended to uncover the regulatory mechanism between Propofol and miR‐520a‐3p in HCC cells.

LIM domain kinase 1 (LIMK1) is a member of LIM kinases. LIM kinases could regulate cell motility through modulating actin dynamics.^20^ Su *et al*. reported that downregulation of LIMK1 suppressed the metastasis of colon cancer.^21^ Li *et al*. demonstrated that miR‐200b‐3p and miR‐429‐5p could block the growth and metastasis of breast cancer cells via downregulating LIMK1/CFL1 signaling.^22^ Nevertheless, the mechanism of LIMK1 in HCC remains to be revealed.

We found that Propofol‐treated Huh7 cells could downregulate the level of H19 in highly metastatic MHCC97‐H and HCCLM3 cells and inhibit the metastasis of the two cells. The underlying mechanism was then explored.

## 2 MATERIALS AND METHODS

### 2.1 Cell culture and Propofol treatment

Three HCC cell lines, including conventional hepatocellular carcinoma cell line (Huh7) and two highly metastatic HCC cell lines (MHCC97‐H and HCCLM3) were acquired from BeNa Culture Collection (Beijing, China). The three cells were grown in Roswell Park Memorial Institute‐1640 (RPMI‐1640) medium (Gibco, Carlsbad, CA, USA) added with 10% fetal bovine serum (FBS; Gibco), 100 units/mL penicillin/100 μg/mL streptomycin in a 37℃ humidified atmosphere with 5% CO_2_. HCC cells were treated with increased concentrations (0 μmol/L, 5 μmol/L, 25 μmol/L, or 50 μmol/L) of Propofol (Sigma, St. Louis, MO, USA) for 24 hours.

#### 2.1.1 (4,5‐Dimethylthiazol‐2‐yl)‐2,5‐diphenyltetrazolium bromide (MTT) assay

The viability and proliferation of HCC cells were analyzed by MTT assay. Huh7 cells were treated with various concentrations (0 μmol/L, 5 μmol/L, 25 μmol/L, or 50 μmol/L) of Propofol, and MTT reagent (Invitrogen, Carlsbad, CA, USA) was used to analyze the viability of Huh7 cells. MHCC97‐H and HCCLM3 cells after exosome treatment and transfection for 0 hours, 24 hours, 48 hours, or 72 hours were mixed with 10 µL MTT reagent (Invitrogen), followed by the detection of the absorbance at 490 nm on a microplate reader (Bio‐Rad, Hercules, CA, USA).

### 2.2 Quantitative real‐time polymerase chain reaction (qRT‐PCR)

RNA sample was isolated using RNAiso (Takara, Otsu, Japan). SYBR‐Green qPCR Mix (Bio‐Rad) was used to amplify template DNA. The abundance of H19, miR‐520a‐3p and LIMK1 was analyzed using the formula of 2^−ΔΔCt^ and normalized to U6 small nuclear RNA or glyceraldehyde‐3‐phosphate dehydrogenase (GAPDH). Relevant primers were listed as follows: H19, Forward (F), 5’‐GATGACAGGTGTGGTCAACG‐3’, Reverse (R), 5’‐CAGACATGAGCTGGGTAGCA‐3’; miR‐520a‐3p, F, 5’‐ACACTCCAGCTGGGAAAGTGCTTCCC‐3’, R, 5’‐CTCAACTGGTGTCGTGGA‐3’; LIMK1, F, 5’‐ATGAGGTTGACGCTACTTTGTTG‐3’, R, 5’‐CTACACTCGCAGCACCTGAA‐3’; U6, F, 5’‐CTCGCTTCGGCAGCACA‐3’, R, 5’‐AACGCTTCACGAATTTGCGT‐3’; GAPDH, F, 5’‐TGGGTGTGAACCACGAGAA‐3’, R, 5’‐GGCATGGACTGTGGTCATGA‐3’.

### 2.3 Western blot assay

HCC cell lysate was centrifuged, and the protein supernatant was quantified using BCA Protein Assay kit (Thermo Fisher Scientific, Waltham, MA, USA). Equal amounts of protein samples (35 μg for protein detection of cell lysate; 20 μg for the detection of exosome markers) were separated using sodium dodecyl sulfate polyacrylamide gel electrophoresis (SDS‐PAGE) and transferred onto polyvinylidene difluoride (PVDF) membranes (Millipore, Billerica, MA, USA). The membranes were incubated with 5% nonfat milk to block the nonspecific sites, followed by incubation with the following primary antibodies at 4℃ overnight. Antibodies against Snail (ab229701; 1:500), Twist (ab175430; 1:1000), Vimentin (ab137321; 1:2000), E‐cadherin (ab40772; 1:40 000), CD9 (ab195422; 1:1000), CD81 (ab109201; 1:5000), LIMK1 (ab81046; 1:1000), and GAPDH (ab181602; 1:10 000) were purchased from Abcam (Cambridge, MA, USA). After washing, the secondary antibody (ab150077, Abcam) was incubated with the membranes. The protein signal was visualized by the enhanced chemiluminescence (ECL) system (Millipore).

### 2.4 Exosome isolation and identification

The culture medium of Huh7 cells was centrifuged at 3000 g for 15 min, and a 0.22 mm filter (Millipore) was used to filter the supernatant. The culture medium was then mixed with Exoquick exosome precipitation solution (System Biosciences, Beijing, China) for 24 hours. After centrifuging at 1500*g* for 30 minutes, the supernatant was discarded. Exosomes were suspended in phosphate buffered saline (PBS) solution for the following experiments. Exosomes were identified by transmission electron microscopy (TEM) and Western blot assay.

### 2.5 Flow cytometry

Annexin‐V‐fluorescein isothiocyanate (FITC) Apoptosis Detection Kit (R&D Systems, Abingdon, UK) was used in this study to detect the apoptosis of HCC cells. HCC cells were suspended in binding buffer followed by dyeing with propidium iodide (PI) and Annexin‐V‐FITC. After washing using PBS, the samples were analyzed through a flow cytometer (BD Biosciences, San Jose, CA, USA).

### 2.6 Transwell assays

Transwell assays were conducted to detect the metastasis of HCC cells. For the detection of cell invasion, the porous membrane was coated with Matrigel (BD Biosciences). HCC cells suspended in 200 μL serum‐free medium were seeded into the precoated upper chambers. The low chambers were filled with 500 μL culture medium supplemented with 10% FBS. The invaded HCC cells were dyed using crystal violet after 24 hours cultivation. The number of invaded HCC cells was counted under a microscope. For the detection of cell migration, the HCC cells were seeded into the noncoated upper chambers, and the other methods were same as above.

### 2.7 Cell transfection

Transfection was conducted with lipofectamine 3000 (Invitrogen). H19 ectopic expression plasmid (Over H19), LIMK1 ectopic expression plasmid (Over LIMK1), empty vector (Vector), miR‐520a‐3p mimic and its control (mimic NC) were obtained from Genepharma (Shanghai, China).

### 2.8 Dual‐luciferase reporter assay

The target relationship between miR‐520a‐3p and H19 or LIMK1 was validated by dual‐luciferase reporter assay. The sequences of H19, including the mutant or wild‐type binding sites of miR‐520a‐3p, were cloned into pSI‐check2 vector (Promega, Madison, WI, USA), termed as H19‐WT or H19‐MUT. 293T cells were co‐transfected with mimic NC or miR‐520a‐3p mimic and H19‐WT or H19‐MUT. Dual‐luciferase reporter assay system (Promega) was utilized to measure the luciferase activity after transfection for 48 hours.

The binding relationship between miR‐520a‐3p and LIMK1 was also confirmed using the same experimental procedure.

### 2.9 Animal experiment

This study was performed with the agreement of the Animal Research Committee of The First Affiliated Hospital of Zhengzhou University. The right flank of BALB/c nude mice (Orient Bio Inc, Seongnam, South Korea; n = 12) was injected with 1 × 10^6^ Huh7 cells. The mice were randomly divided into two groups (n = 6 in each group) when the tumor volume reached about 100 mm^3^ (Day 10). The two groups were injected with exosomes (30 μg) derived from Huh7 cells treated with Propofol and Vector or Over H19, respectively. Meanwhile, Propofol (35 mg/kg) was intraperitoneally injected to the mice every 2 d. The size of tumors was assessed every 4 d with the formula of volume = π/6 × length ×width × height. The weight of tumors was recorded after inoculation for 34 d. qRT‐PCR and Western blot were performed to detect the expression of H19, miR‐520a‐3p, LIMK1, and metastasis‐associated proteins in resected tumor tissues.

### 2.10 Statistical analysis

Statistical analysis of the data from three independent experiments was conducted using GraphPad Prism 7. All data were displayed as mean ± standard deviation (SD). *P* value was analyzed using Student's *t*‐test or one‐way analysis of variance (ANOVA) followed by Tukey's test. *P* < 0.05 was indicated as statistically significant.

## 3 RESULTS

### 3.1 The expression of H19 is downregulated in HCC cells co‐cultured with Huh7 cells treated with Propofol

**Figure 1:**
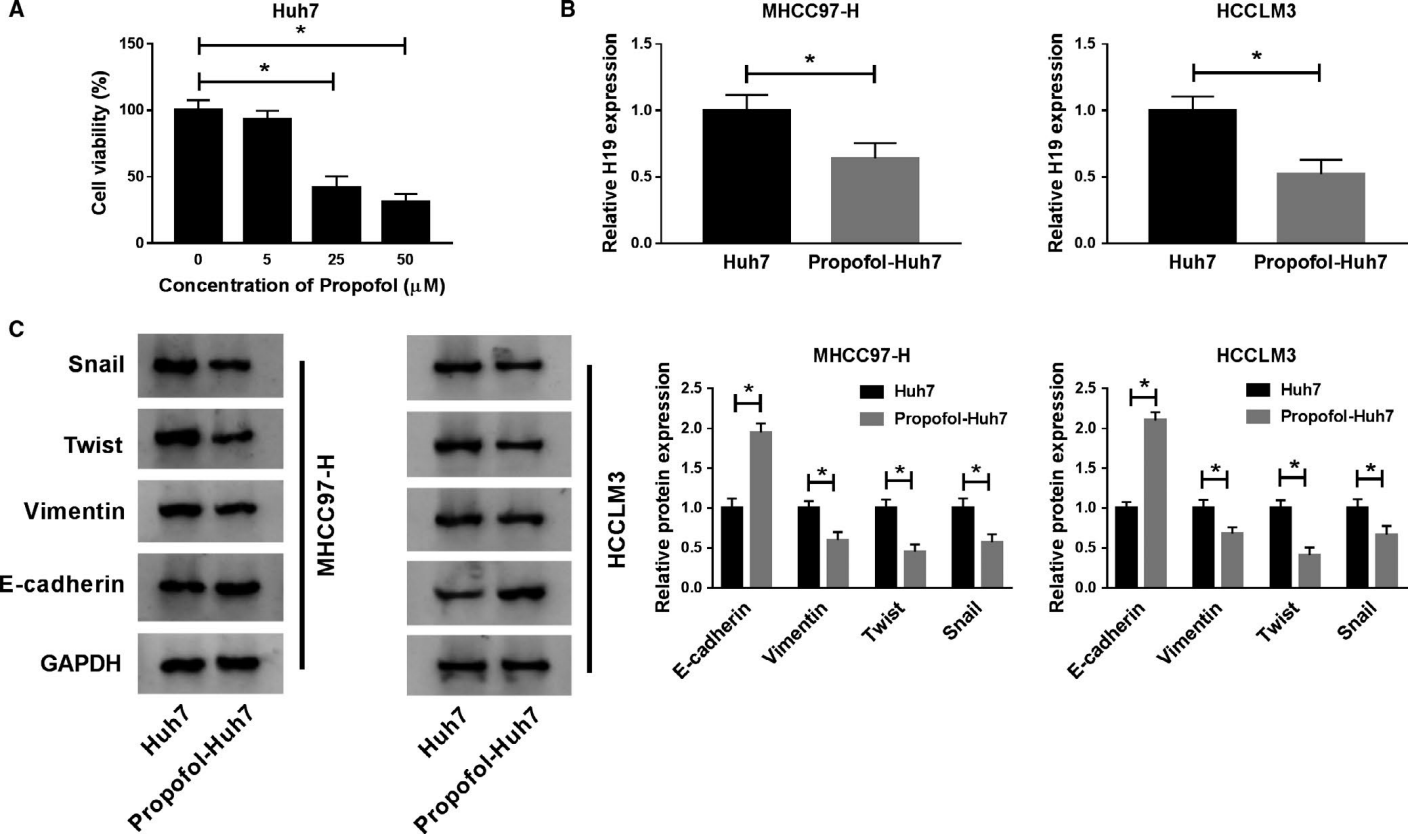
The expression of H19 is downregulated in HCC cells co‐cultured with Huh7 cells treated with Propofol. (A) The viability of Huh7 cells treated with diverse concentrations of Propofol (0 μmol/L, 5 μmol/L, 25 μmol/L, or 50 μmol/L) was detected by MTT assay. (B and C) MHCC97‐H and HCCLM3 cells were co‐cultured with Huh7 cells treated with Propofol (25 μmol/L) or not. (B) The expression of H19 was measured in MHCC97‐H and HCCLM3 cells by qRT‐PCR. (C) The abundance of metastasis‐associated proteins (Snail, Twist, Vimentin, and E‐cadherin) was determined in the two HCC cells by Western blot assay. *P < .05

The effect of Propofol on the viability of Huh7 cells was explored by MTT assay. As mentioned in Figure 1A, the viability was markedly decreased in Huh7 cells treated with 25 μmol/L or 50 μmol/L Propofol, while there was no significant change in the viability of Huh7 cells treated with 5 μmol/L Propofol compared with nontreated Huh7 cells. We chose two highly metastatic HCC cell lines (MHCC97‐H and HCCLM3 cells) to investigate the influence of Propofol. As indicated in Figure 1B, the expression of H19 was lower in MHCC97‐H and HCCLM3 cells cocultured with Huh7 cells treated with Propofol than that in HCC cells co‐cultured with nontreated Huh7 cells. Besides, the cocultivation with Propofol‐Huh7 cells reduced the expression of Vimentin, Twist, and Snail in MHCC97‐H and HCCLM3 cells, while the level of E‐cadherin was upregulated (Figure 1C). Taken together, Propofol‐treated Huh‐7 decreased the abundance of H19 in MHCC97‐H and HCCLM3 cells, and the metastasis of the two HCC cells was also inhibited.

### 3.2 Propofol treatment reduces the expression of H19 in the exosomes of Huh7 cells

**Figure 2:**
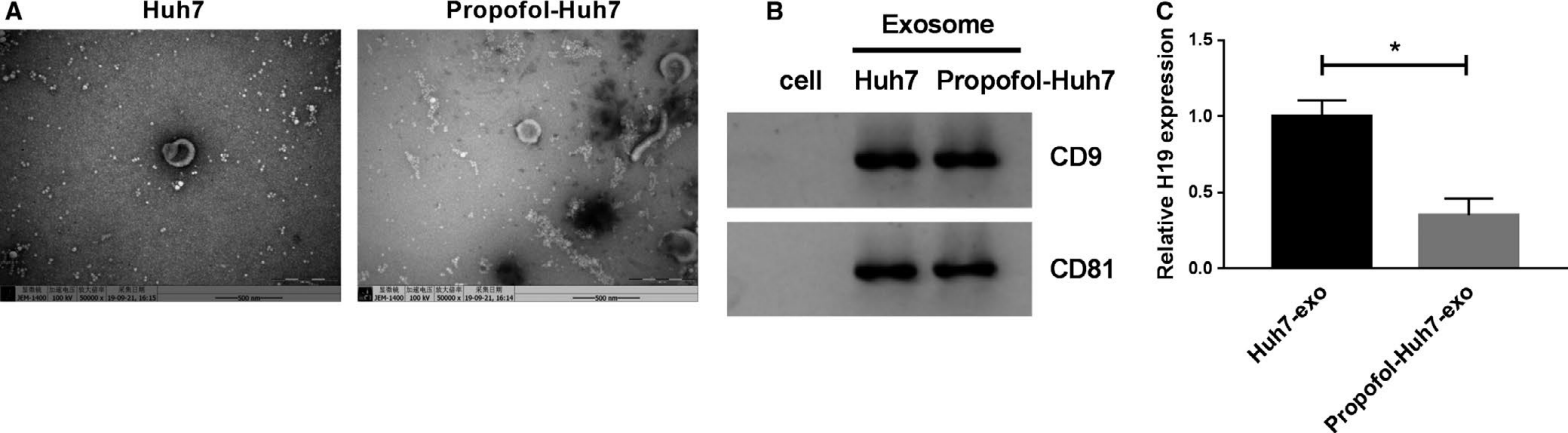
Propofol treatment reduces the expression of H19 in the exosomes of Huh7 cells. (A) The exosomes from Huh7 cells treated with Propofol (25 μmol/L) or not were isolated, and then the exosomes were photographed using TEM. (B) The markers of exosomes, including CD9 and CD81, were detected by Western blot assay. (C) The level of H19 was examined in exosomes generated from Huh7 cells treated with Propofol (25 μmol/L) or not by qRT‐PCR. *P < .05

To explore whether exosomes were involved in the modulation of Huh7 cells on MHCC97‐H and HCCLM3 cells, we extracted exosomes from the culture medium of Huh7 and Propofol‐treated Huh7 cells. The exosomes were characterized by TEM and Western blot assay. The lipid bilayer membrane structure of exosomes from Huh7 and Propofol‐treated Huh7 cells was shown in Figure 2A. Western blot results showed that two exosome markers (CD9 and CD81) were detectable in exosomes from Huh7 and Propofol‐treated Huh7 cells instead of cell lysate (Figure 2B). There was a notable decrease in the expression of H19 in Propofol‐Huh7‐exo in comparison with that in Huh7‐exo (Figure 2C). These findings implied that exosomal H19 might exert a crucial role in the modulation of MHCC97‐H and HCCLM3 cells.

### 3.3 Exosomes from Huh7 cells treated with Propofol inhibit the proliferation, metastasis and promotes the apoptosis of HCC cells

**Figure 3:**
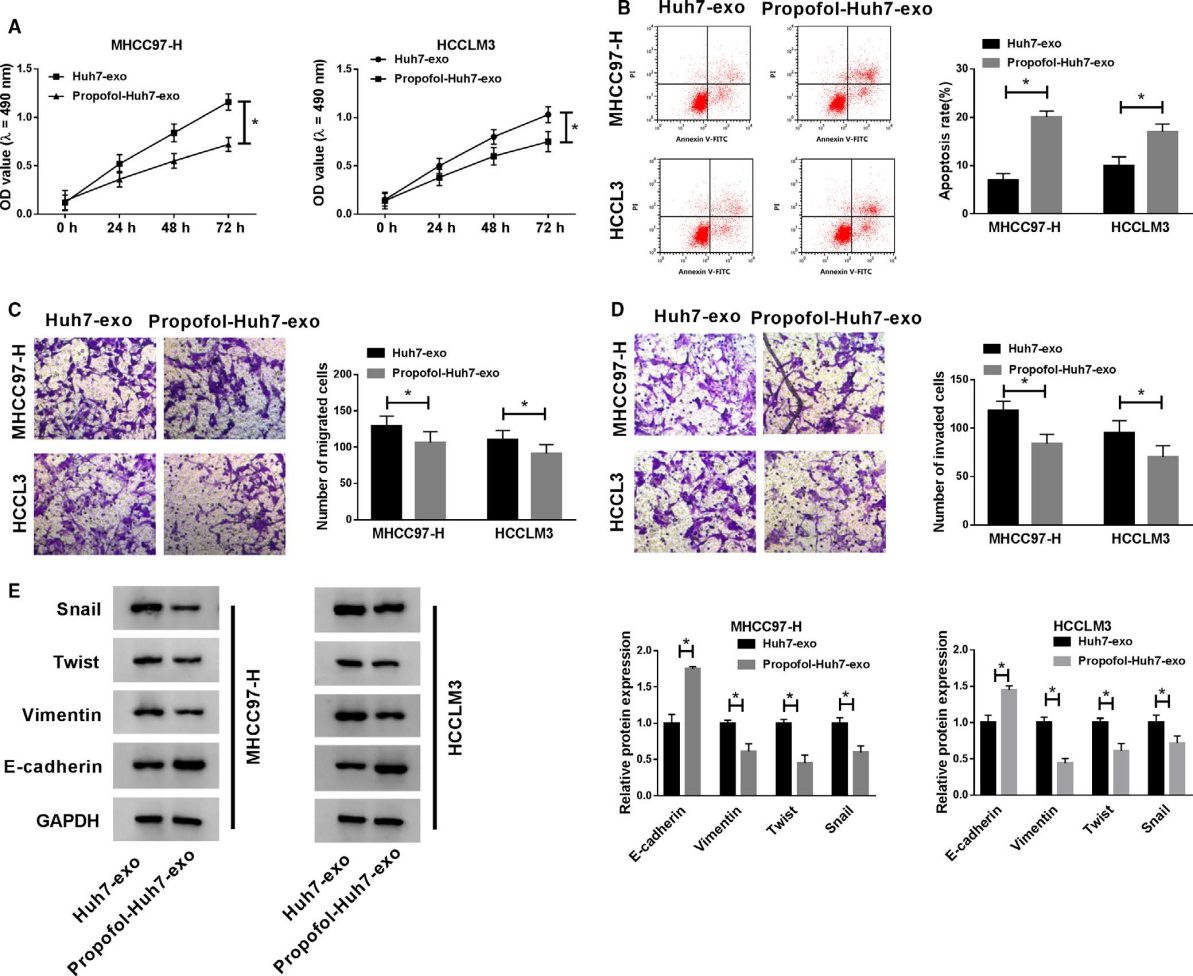
Exosomes from Huh7 cells treated with Propofol inhibits the proliferation, metastasis, and promotes the apoptosis of HCC cells. MHCC97‐H and HCCLM3 cells were incubated with exosomes from Huh7 cells exposed to Propofol (25 μmol/L) or not. (A) MTT assay was performed to examine the proliferation of HCC cells. (B) Flow cytometry was carried out to detect the apoptosis of HCC cells. (C and D) Transwell assays were conducted to detect the migration and invasion of HCC cells. (E) Western blot was conducted to detect the expression of Snail, Twist, Vimentin, and E‐cadherin in HCC cells. *P < .05

MHCC97‐H and HCCLM3 cells were incubated with Huh7‐exo or Propofol‐Huh7‐exo. As mentioned in Figure 3A, the proliferation of MHCC97‐H and HCCLM3 cells was conspicuously restrained in Propofol‐Huh7‐exo group than that in Huh7‐exo group. Moreover, the addition of exosomes generated from Propofol‐treated Huh7 cells significantly promoted the apoptosis of MHCC97‐H and HCCLM3 cells (Figure 3B). The migration and invasion of MHCC97‐H and HCCLM3 cells were blocked in Propofol‐Huh7‐exo group compared with Huh7‐exo group (Figure 3C and D). We further examined the protein levels of Snail, Twist, Vimentin, and E‐cadherin to confirm the effect of Propofol‐Huh7‐exo on the metastasis of MHCC97‐H and HCCLM3 cells. The enrichment of Snail, Twist, and Vimentin was declined while E‐cadherin was upregulated in HCC cells incubated with exosomes derived from Propofol‐exposed Huh7 cells (Figure 3E). In summary, Huh7 cells treated with Propofol could inhibit the malignant potential of highly metastatic HCC cells via exosomes.

### 3.4 Exosomal H19 from Huh7 cells enhances the malignant potential of HCC cells

**Figure 4:**
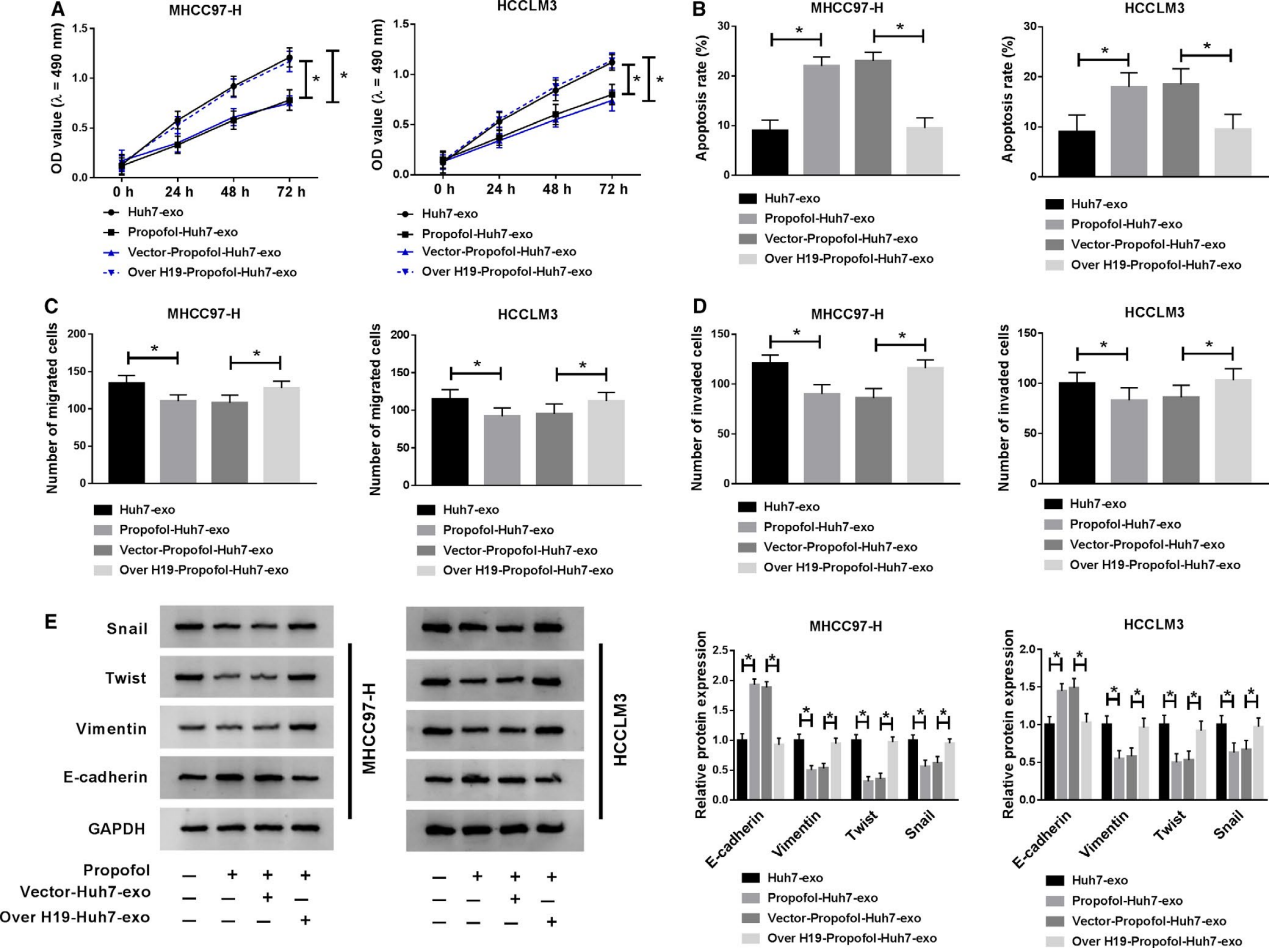
Exosomal H19 from Huh7 cells enhances the malignant potential of HCC cells. Huh7 cells were treated with Propofol, Vector + Propofol or Over H19 + Propofol, and exosomes were fractionated from the above cells and untreated Huh7 cells. MHCC97‐H and HCCLM3 cells were incubated with exosomes for 24 h. (A) The proliferation of MHCC97‐H and HCCLM3 cells was determined through performing MTT assay. (B) Flow cytometry was applied to detect the apoptosis of MHCC97‐H and HCCLM3 cells. (C and D) The numbers of migrated and invaded HCC cells were counted through transwell assays. (E) Metastasis‐associated markers were measured in HCC cells by Western blot assay. *P < .05

We wondered whether H19 in exosomes exerted a pivotal role in the regulation of the behaviors of HCC cells, and rescue experiments were conducted. As indicated in Figure 4A and B, the exosomes from Huh7 cells treated with H19 overexpression plasmid and Propofol could attenuate the inhibitory effect of Propofol‐Huh7‐exo on the proliferation and the promoting impact on the apoptosis of HCC cells. Also, the migration and invasion of HCC cells were recovered in Over H19‐Propofol‐Huh7‐exo group (Figure 4C and D). The changes in the expression of metastasis‐related proteins also suggested that the inhibitory effect of Propofol‐Huh7‐exo addition on the metastasis of HCC cells was alleviated by the addition of Over H19‐Propofol‐Huh7‐exo (Figure 4E). These findings demonstrated that the depletion of exosomal H19 caused by Propofol treatment in Huh7 cells inhibited the malignant potential of HCC cells.

### 3.5 MiR‐520a‐3p is a target of H19

**Figure 5:**
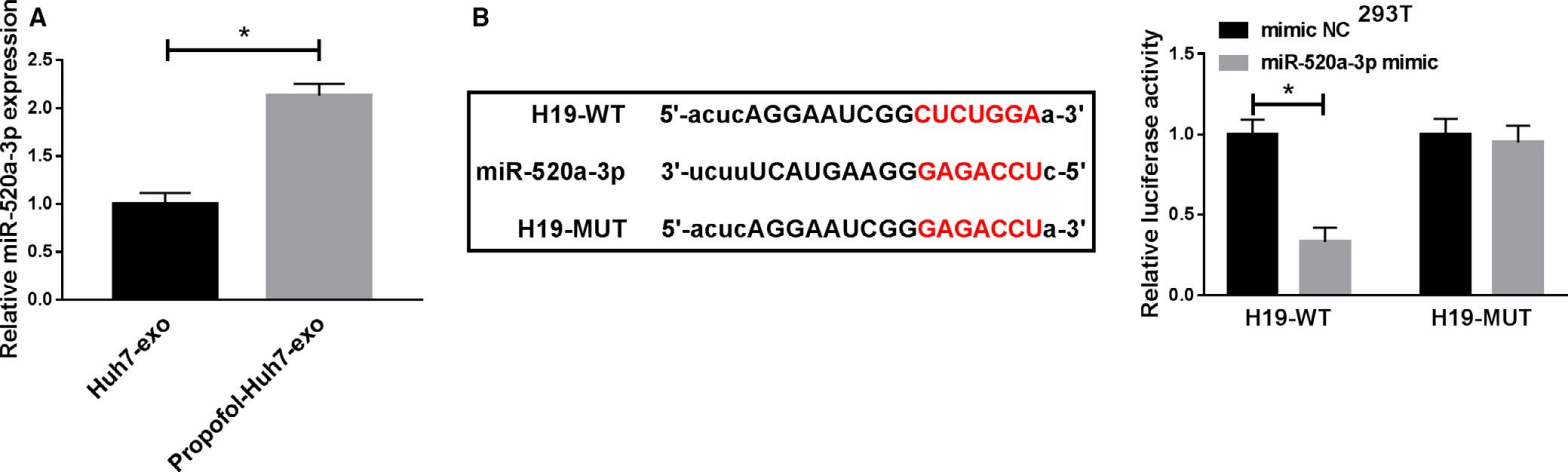
MiR‐520a‐3p is a target of H19. (A) The enrichment of miR‐520a‐3p was examined in the exosomes generated from Huh7 cells treated with Propofol or not by qRT‐PCR. (B) The interaction between miR‐520a‐3p and H19 was predicted by StarBase software, and the interaction was verified by dual‐luciferase reporter assay. *P < .05

The expression of miR‐520a‐3p was markedly enhanced in exosomes generated from Propofol‐treated Huh7 cells other than nontreated Huh7 cells (Figure 5A). Furthermore, miR‐520a‐3p was a candidate target gene of H19 predicted by StarBase software (Figure 5B), and the cotransfection of miR‐520a‐3p mimic and H19‐WT reduced the luciferase activity in 293T cells compared with that in mimic NC and H19‐WT group. In summary, miR‐520a‐3p could directly bind to H19.

### 3.6 Exosomal H19 from Huh7 cells promotes the proliferation and metastasis and impedes the apoptosis of HCC cells via sponging miR‐520a‐3p

**Figure 6:**
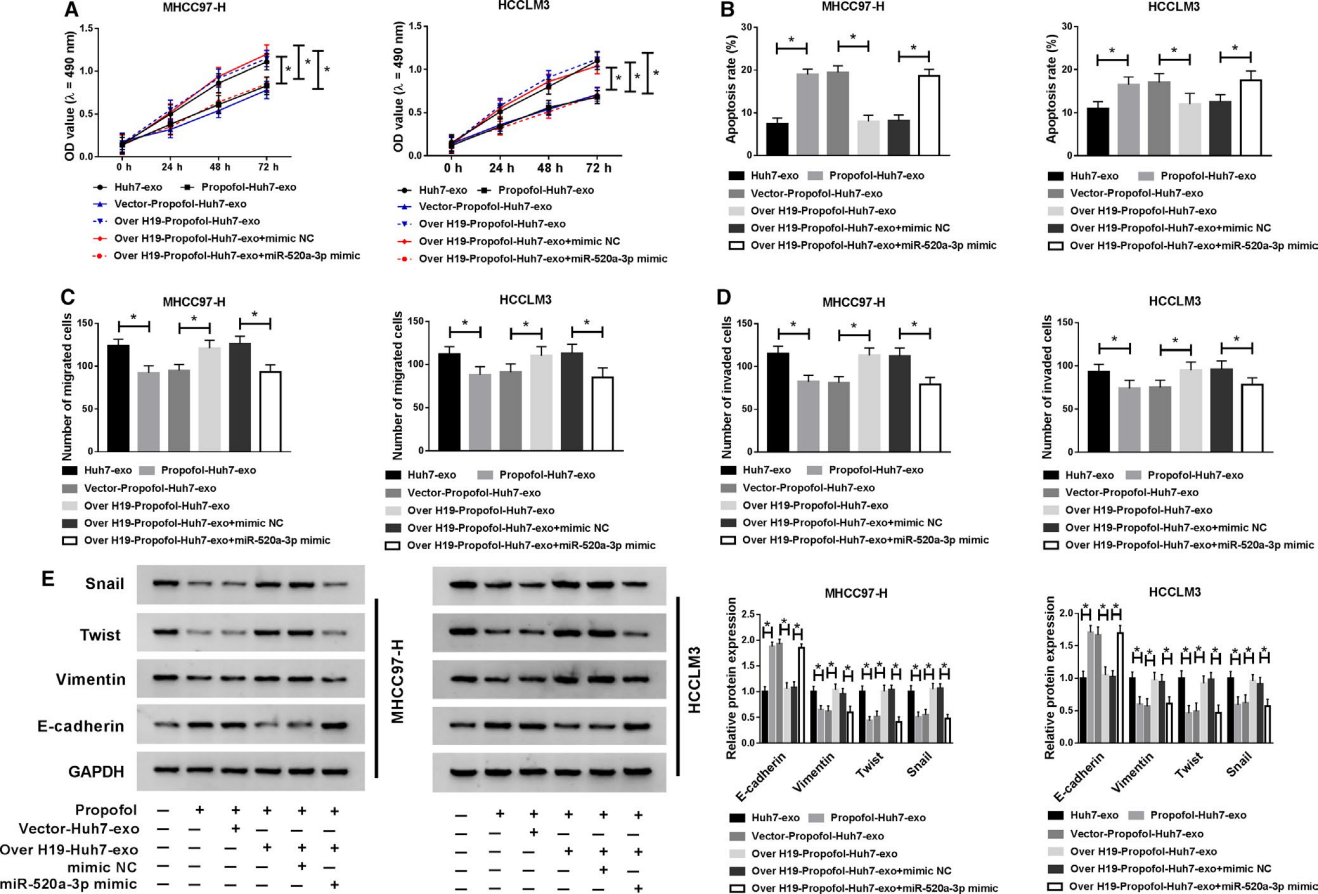
Exosomal H19 from Huh7 cells promotes the proliferation and metastasis and impedes the apoptosis of HCC cells via sponging miR‐520a‐3p. MHCC97‐H and HCCLM3 cells were treated with Huh7‐exo, Propofol‐Huh7‐exo, Vector‐Propofol‐Huh7‐exo, Over H19‐Propofol‐Huh7‐exo, Over H19‐Propofol‐Huh7‐exo + mimic NC or Over H19‐Propofol‐Huh7‐exo + miR‐520a‐3p mimic. (A) The proliferation of HCC cells was determined through MTT assay. (B) The apoptosis of HCC cells was analyzed by flow cytometry. (C and D) The abilities of migration and invasion of HCC cells were measured through conducting transwell assays. (E) Western blot assay was applied to detect the abundance of metastasis‐related proteins in HCC cells. *P < .05

MHCC97‐H and HCCLM3 cells were treated with Huh7‐exo, Propofol‐Huh7‐exo, Vector‐Propofol‐Huh7‐exo, Over H19‐Propofol‐Huh7‐exo, Over H19‐Propofol‐Huh7‐exo + mimic NC or Over H19‐Propofol‐Huh7‐exo + miR‐520a‐3p mimic to uncover the relationship between miR‐520a‐3p and H19. As mentioned in Figure 6A and B, the overexpression of miR‐520a‐3p diminished the promoting effect of Over H19‐Propofol‐Huh7‐exo on the proliferation and the suppressive influence on the apoptosis of HCC cells. The results of transwell assays and Western blot revealed that the upregulation of metastasis in HCC cells caused by the addition of Over H19‐Propofol‐Huh7‐exo was counteracted by the transfection of miR‐520a‐3p mimic (Figure 6C‐E). Collectively, the high expression of circulating H19 accelerated the progression of HCC via sponging miR‐520a‐3p.

### 3.7 LIMK1 could bind to miR‐520a‐3p

**Figure 7:**
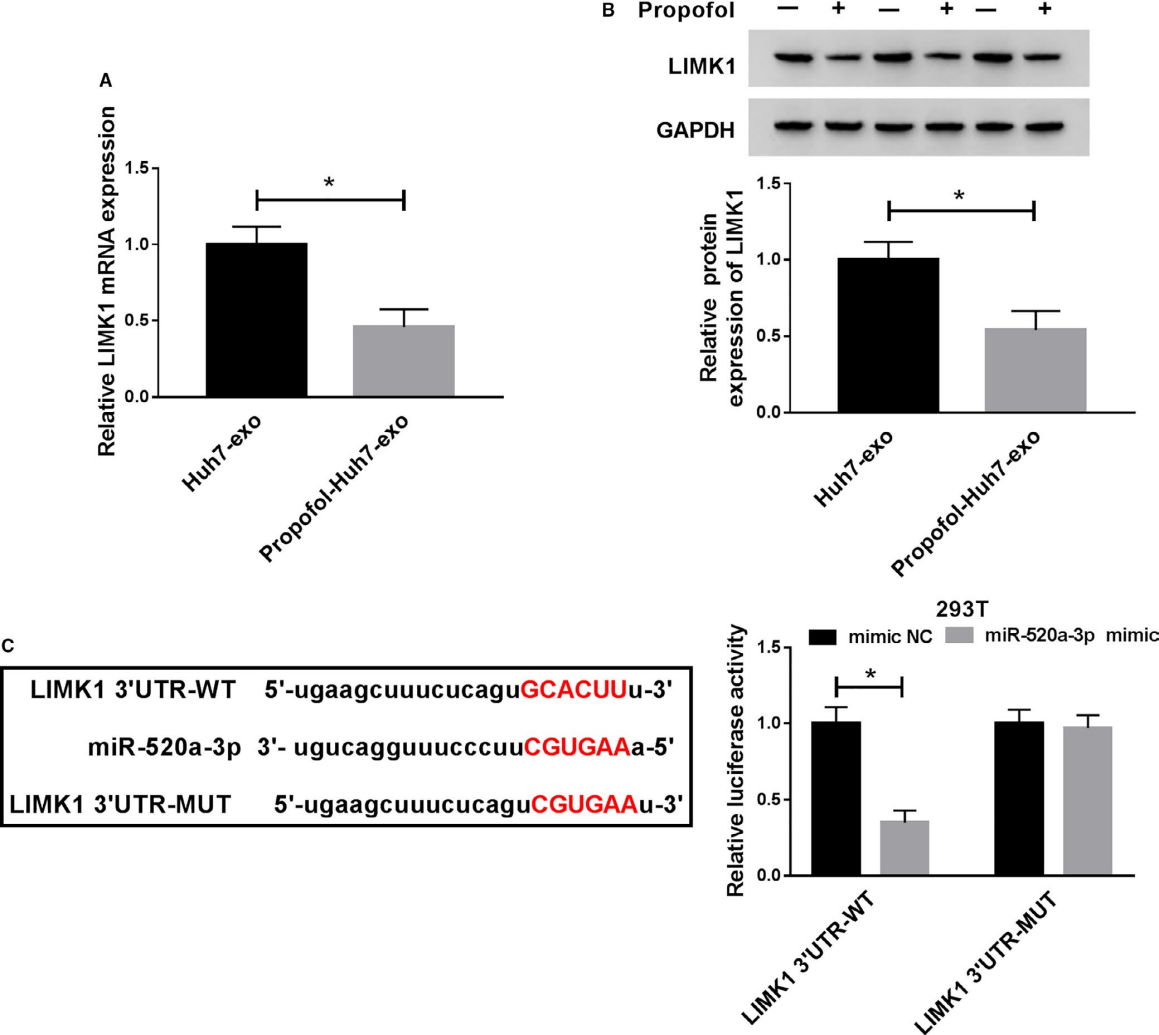
LIMK1 could bind to miR‐520a‐3p. (A and B) The mRNA and protein abundance of LIMK1 was detected in exosomes derived from Huh7 cells treated with Propofol or not by qRT‐PCR and Western blot assay. (C) The binding sites between miR‐520a‐3p and LIMK1 were predicted using StarBase bioinformatic database, and dual‐luciferase reporter assay was applied to confirm this target relationship in 293T cells. *P < .05

Propofol treatment also declined the mRNA and protein expression of LIMK1 in Huh7 exosomes (Figure 7A and B). StarBase database predicted that there were binding sites between the 3’ untranslated region (3’UTR) of LIMK1 and miR‐520a‐3p (Figure 7C), and a notable decrease of the luciferase activity was observed in miR‐520a‐3p and LIMK1 3’UTR‐WT cotransfected group in contrast to that in mimic NC and LIMK1 3’UTR‐WT group. Besides, the luciferase activity was unaffected in LIMK1 3’UTR‐MUT and mimic NC or miR‐520a‐3p group. Taken together, miR‐520a‐3p could bind to the 3’UTR of LIMK1.

### 3.8 The accumulation of miR‐520a‐3p reverses the effects of exosomal LIMK1 on HCC cells

**Figure 8:**
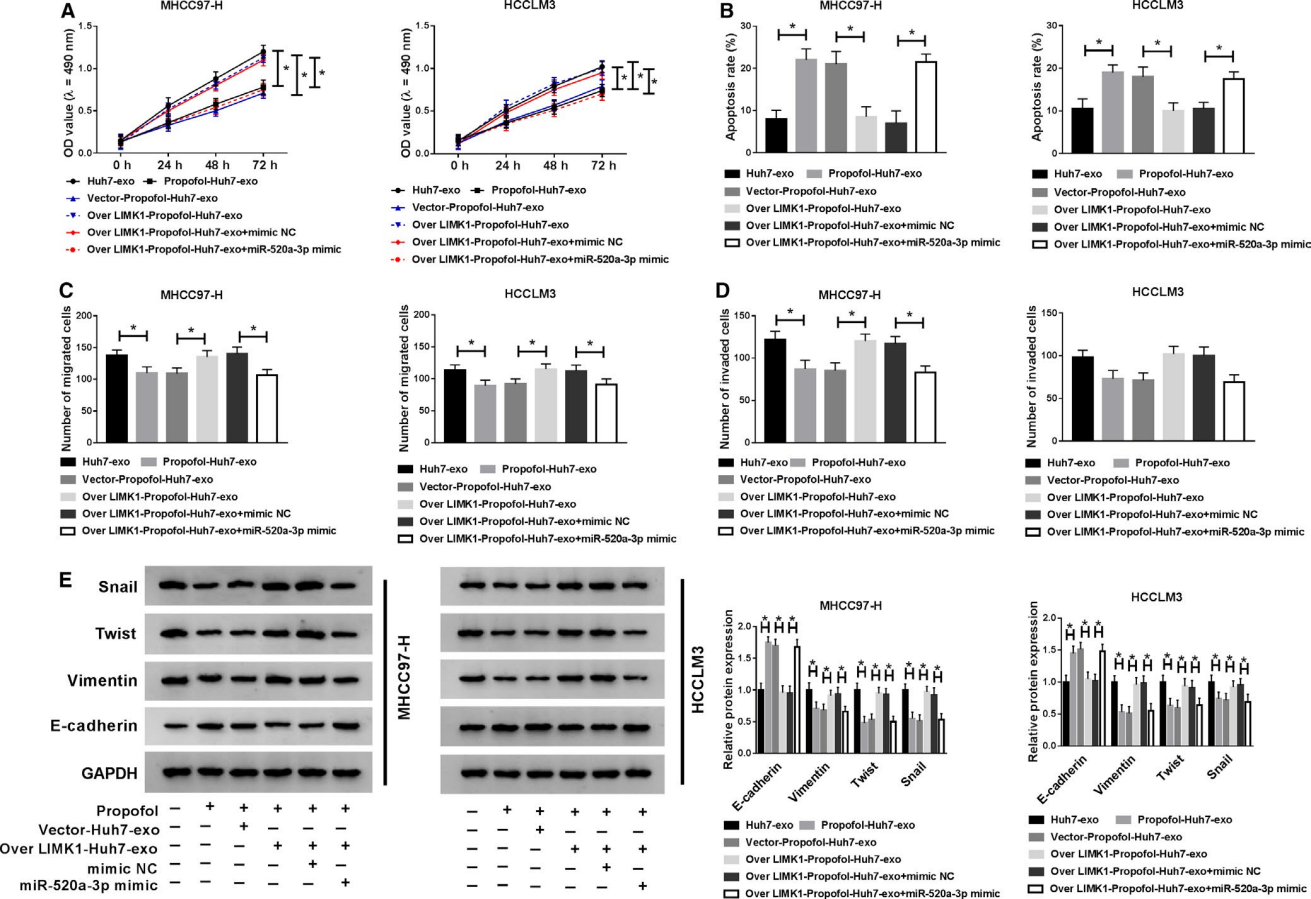
The accumulation of miR‐520a‐3p reverses the effects of exosomal LIMK1 on HCC cells. MHCC97‐H and HCCLM3 cells were treated with Huh7‐exo, Propofol‐Huh7‐exo, Vector‐Propofol‐Huh7‐exo, Over LIMK1‐Propofol‐Huh7‐exo, Over LIMK1‐Propofol‐Huh7‐exo + mimic NC or Over LIMK1‐Propofol‐Huh7‐exo + miR‐520a‐3p mimic. (A) MTT assay was conducted to detect the proliferation of HCC cells. (B) Flow cytometry was used to detect the apoptosis rate of HCC cells. (C and D) Transwell assays were performed to examine the metastasis of HCC cells. (E) The enrichment of metastasis‐related proteins in HCC cells was examined by Western blot assay. *P < .05

The high expression of LIMK1 in exosomes facilitated the proliferation and restrained the apoptosis of HCC cells, and the co‐treatment with miR‐520a‐3p mimic counteracted the effects caused by the addition of Over LIMK1‐Propofol‐Huh7‐exo (Figure 8A and B). The motility of HCC cells was promoted with the addition of Over LIMK1‐Propofol‐Huh7‐exo, and the addition of miR‐520a‐3p hampered the motility of HCC cells again (Figure 8C‐E). These results suggested that miR‐520a‐3p inhibited the development of HCC through LIMK1.

### 3.9 Exosomal H19 upregulates the expression of LIMK1 in HCC cells through suppressing miR‐520a‐3p

**Figure 9:**
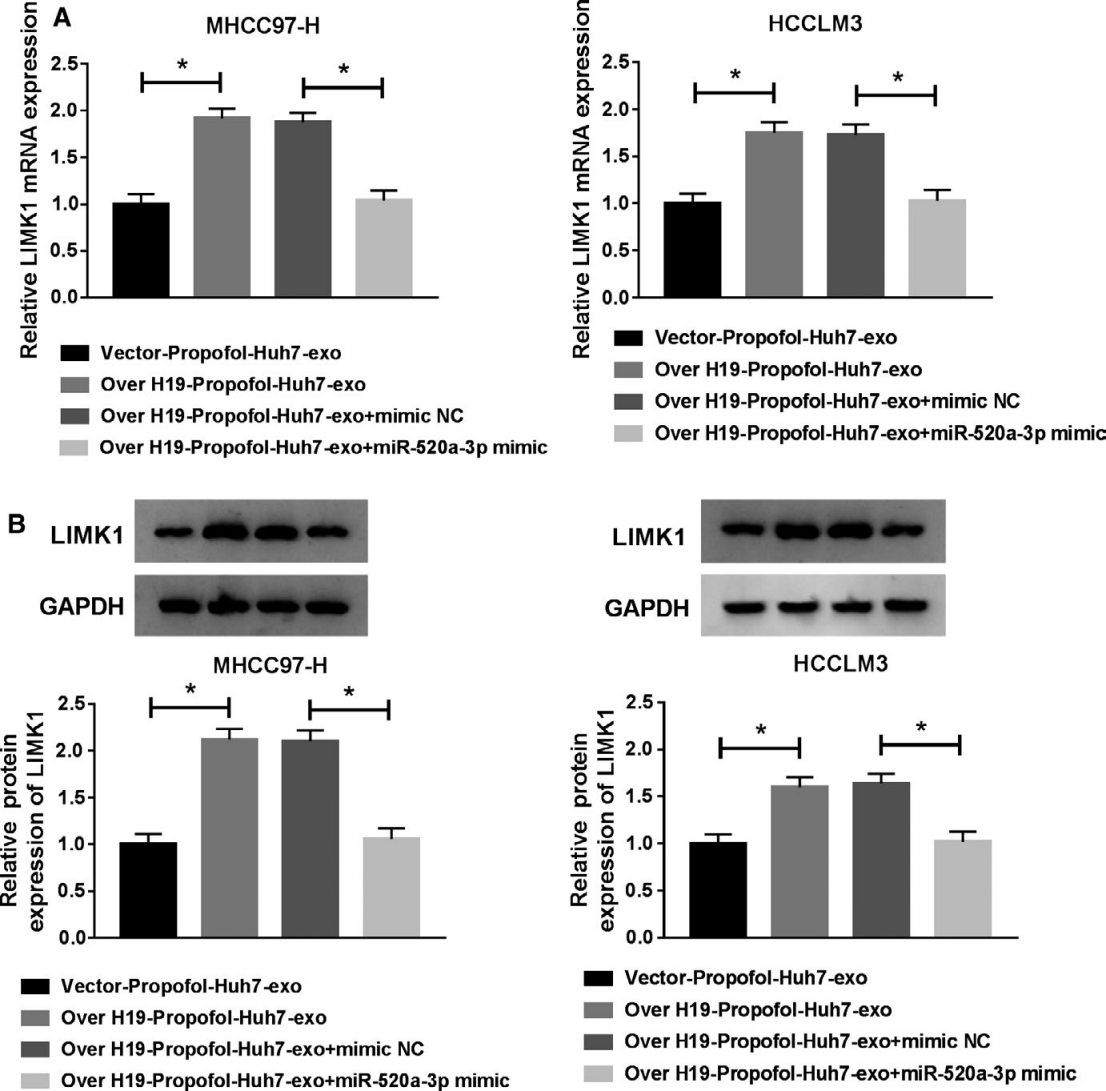
Exosomal H19 upregulates the expression of LIMK1 in HCC cells through suppressing miR‐520a‐3p. MHCC97‐H and HCCLM3 cells were treated with Vector‐Propofol‐Huh7‐exo, Over H19‐Propofol‐Huh7‐exo, Over H19‐Propofol‐Huh7‐exo + mimic NC or Over H19‐Propofol‐Huh7‐exo + miR‐520a‐3p mimic. (A and B) qRT‐PCR and Western blot assay were utilized to detect the levels of LIMK1 mRNA and protein in HCC cells. *P < .05

To further elucidate the regulatory relationship among H19, miR‐520a‐3p and LIMK1, we treated HCC cells with Over H19‐Propofol‐Huh7‐exo + miR‐520a‐3p mimic. The addition of Over H19‐Propofol‐Huh7‐exo elevated the mRNA and protein expression of LIMK1 in HCC cells, and the abundance of LIMK1 mRNA and protein was repressed with the transfection of miR‐520a‐3p mimic (Figure 9A and B). In summary, LIMK1 was modulated by H19/miR‐520a‐3p axis in HCC cells.

### 3.10 Exosomal H19 promotes the growth of HCC tumors treated with Propofol

**Figure 10:**
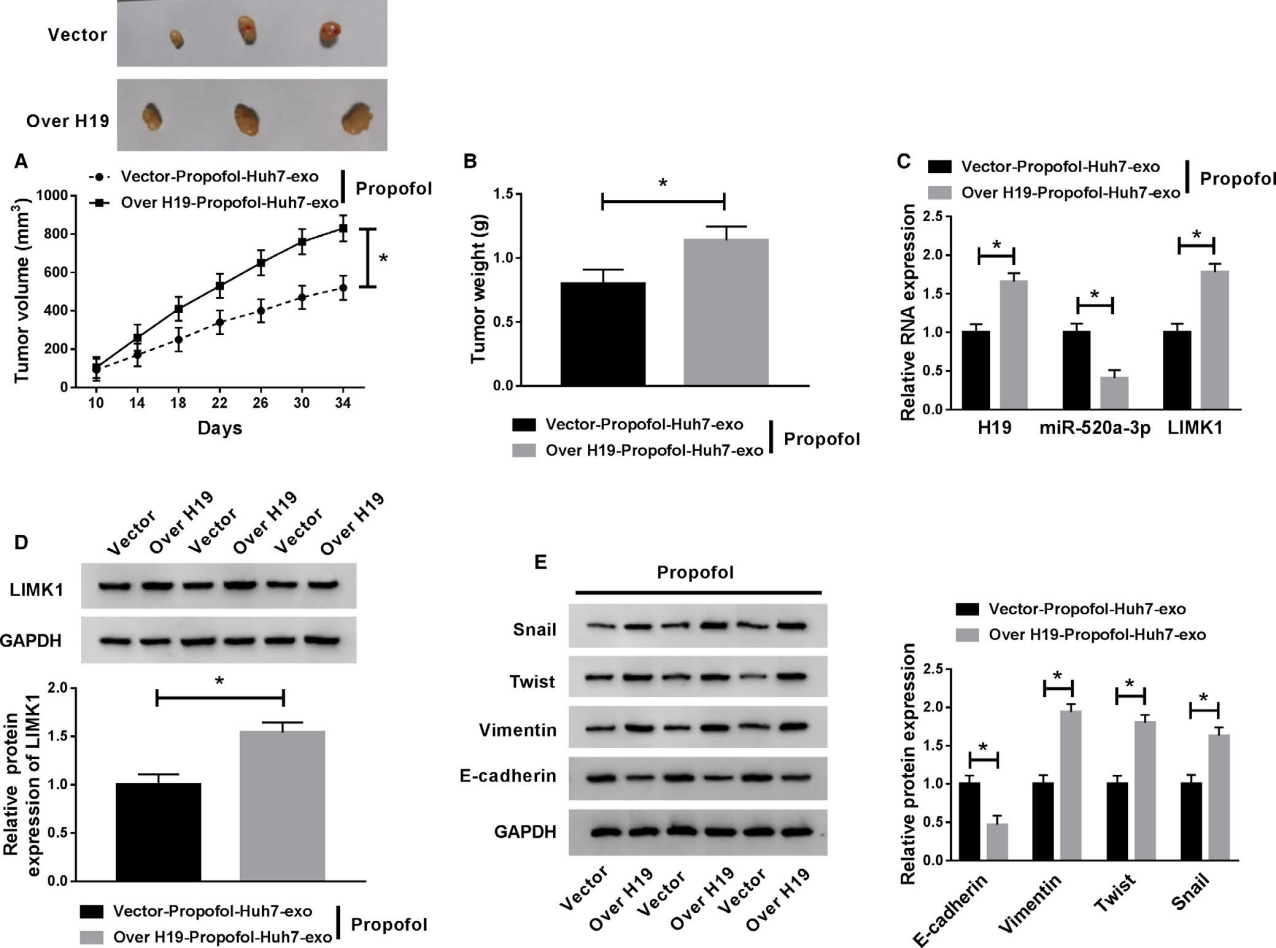
Exosomal H19 promotes the growth of HCC tumors treated with Propofol. (A) The dimension of HCC tumors was measured every 4 d. (B) The HCC tumors were resected from BALB/c nude mice after injection for 34 d, and the weight of tumors was recorded. (C) qRT‐PCR was carried out to measure the RNA expression of H19, miR‐520a‐3p and LIMK1 in tumor tissues. (D) The protein level of LIMK1 was detected in tumor tissues by Western blot assay. (E) The expression of Snail, Twist, Vimentin, and E‐cadherin in tumor tissues was examined by Western blot assay. *P < .05

The volume and weight of HCC tumors were higher in Over H19‐Propofol‐Huh7‐exo + Propofol group than that in Vector‐Propofol‐Huh7‐exo + Propofol group (Figure 10A and B). To verify whether these effects were due to the abnormal expression of H19, miR‐520a‐3p and LIMK1, qRT‐PCR and Western blot were performed. As mentioned in Figure 10C and D, H19 and the mRNA and protein expression of LIMK1 were upregulated with the addition of Over H19‐Propofol‐Huh7‐exo and Propofol, while the change in the expression of miR‐520a‐3p revealed a reverse phenomenon. Next, we found the expression of Snail, Twist and Vimentin was enhanced in Over H19‐Propofol‐Huh7‐exo and Propofol group, and a decrease in the expression of E‐cadherin was found in this group (Figure 10E). Taken together, high expression of exosomal H19 promotes the growth and metastasis of HCC tumors treated with Propofol.

## 4 DISCUSSION

Propofol has been reported to inhibit the progression of HCC.^23^, ^24^, ^25^ Besides, Bai *et al*. reported that Propofol treatment hampered the metastasis of breast cancer cells through reducing the enrichment of H19.^16^ The expression of lncRNA H19 was declined in MHCC97‐H and HCCLM3 cells co‐culturing with Propofol‐treated Huh7 cells. Meanwhile, the metastasis of MHCC97‐H and HCCLM3 cells was blocked. We wondered if exosomes functioned in the above biological possesses.

Exosomes could be released under physiological and pathological conditions. The communication between tumor cells or stromal cells and tumor cells could be achieved through the transferring of exosomes.^26^, ^27^, ^28^ The small molecules wrapped in exosomes, including lncRNAs and miRNAs, could alter the expression of their target genes in recipient cells, thus modulating the behaviors of recipient cells.^29^ The exosomes generated from untreated Huh7 cells or Propofol‐exposed Huh7 cells were isolated, and we found that the expression of H19 was lower in the exosomes of Huh7 cells treated with Propofol. To confirm the role of exosomes on the behaviors of MHCC97‐H and HCCLM3 cells, we conducted the following experiments. The results revealed that Propofol‐Huh7‐exo inhibited the proliferation, migration, and invasion while promoted the apoptosis of HCC cells.

We wondered whether lncRNA H19 in exosomes regulated the biological functions of HCC cells, and rescue experiments were performed to verify our hypothesis. The low expression of lncRNA H19 in Propofol‐Huh7‐exo group inhibited the malignant potential of HCC cells, while the high abundance of H19 in Over H19‐Propofol‐Huh7‐exo group recovered the malignant potential of HCC cells.

StarBase online software was used to search the downstream targets of lncRNA H19, miR‐520a‐3p was identified as a direct target of lncRNA H19. Yu *et al*. reported that miR‐520a‐3p suppressed the progression of nonsmall cell lung cancer.^30^ Bi *et al*. proved that miR‐520a‐3p blocked the metastasis of papillary thyroid carcinoma cells via JAK/STAT pathway.^31^ The relationship between miR‐520a‐3p and Propofol or lncRNA H19 in HCC has not been reported. We found that the overexpression of miR‐520a‐3p in MHCC97‐H and HCCLM3 cells reversed the effects of the addition of Over H19‐Propofol‐Huh7‐exo, and these findings demonstrated that exosomal H19 exerted its oncogenic role in HCC cells through sponging miR‐520a‐3p.

LIMK family was important modulator of the actin cytoskeleton, and the abnormal expression of LIMK family was related to the progression of metastatic diseases.^32^, ^33^ LIMK1 was confirmed as a target of miR‐520a‐3p. Yu *et al*. demonstrated that sevoflurane treatment mediated learning and memory impairments of rats through upregulating miR‐27b and downregulating LIMK1.^34^ In the current study, we found that Propofol stimulation decreased the expression of LIMK1 in exosomes. Mechanistically speaking, miR‐520a‐3p inhibited the progression of HCC through downregulating LIMK1. Moreover, circulating lncRNA H19 enhanced the enrichment of LIMK1 via sponging miR‐520a‐3p in HCC cells. The results of animal experiment showed that exosomal H19 could promote the growth and metastasis of HCC tumors treated with Propofol in vivo.

## 5 CONCLUSION

Taken together, exosomal lncRNA H19 elevated the malignant potential of HCC cells treated with Propofol via miR‐520a‐3p/LIMK1 axis in vivo and in vitro. The underlying mechanism of LIMK1 on the modulation of behaviors of HCC cells needs further exploration.

## CONFLICT OF INTEREST

The authors declare that they have no competing interests.

## 6 Ethics Statement

All the patients involved in this study signed informed consents. This study was supported by the ethics committee of The First Affiliated Hospital of Zhengzhou University.

## PATIENT CONSENT FOR PUBLICATION

Not applicable.

## AVAILABILITY OF DATA AND MATERIALS

The analyzed datasets generated during the present study are available from the corresponding author on reasonable request.

## AUTHORS’ CONTRIBUTION

Conceptualization and Methodology: Tao Yang and Junqi Liu; Formal analysis and Data curation: Huaping Zhao, Na Xing and Juan He; Validation and Investigation: Dongmei Wang, Yanqiu Ai and Jianjun Yang; Writing ‐ original draft preparation and Writing ‐ review and editing: Dongmei Wang, Tao Yang and Junqi Liu; Approval of final manuscript: all authors.
